# Clinical outcome of endonasal KTP laser assisted dacryocystorhinostomy

**DOI:** 10.1186/1471-2415-5-2

**Published:** 2005-03-03

**Authors:** Thomas Ressiniotis, Gerasimos M Voros, Vasilios T Kostakis, Sean Carrie, Christopher Neoh

**Affiliations:** 1 Department of Ophthalmology, Royal Victoria Infirmary, Newcastle upon Tyne, United Kingdom; 2 Department of Otorhinolaringology, Freeman Hospital, Newcastle upon Tyne, United Kingdom

## Abstract

**Background:**

To evaluate the clinical outcome of primary endonasal laser assisted dacryocystorhinostomy (ENL-DCR) using the potassium-titanyl-phosphate laser.

**Methods:**

We retrospectively reviewed all primary ENL-DCRs performed within a period of twelve months by the same combined Ophthalmology and Otorhinolaringology team in Freeman Hospital, Newcastle upon Tyne, UK. The main outcome measure for success was resolution or significant improvement of epiphora. Details of surgery, intraoperative and postoperative complications, as well as pathology associated with failure were also studied. Patients were followed up for at least 12 months.

**Results:**

A total of 41 consecutive ENL-DCRs on 29 patients (22 females, 7 males, mean age 75 years) were analysed. All patients had bicanalicular silicone intubation for at least 4 months. The success rate at 12 months postoperatively was 78.1%. Pathology associated with failure included: intranasal pathology (12.2%), mucocele (7.3%), and systemic sarcoidosis (2.4%). No significant intra-operative complications were recorded.

**Conclusion:**

The ENL-DCR with potassium-titanyl-phosphate laser can be considered as a safe and efficient primary procedure for the treatment of nasolacrimal duct obstruction.

## Background

Dacryocystorhinostomy (DCR) is the treatment of choice for patients with chronic stenosis and obstruction of the nasolacrimal duct. External dacryocystorhinostomy (EXT-DCR) was first described by Toti [1,2] in 1904. The endonasal approach was first introduced in 1893 by Caldwell [3,4], but it was inherently limited by poor visibility of endonasal anatomy during surgery. The introduction of high-resolution fiberoptic endoscopes in the late 1980s enabled adequate visualisation of the nasal cavities, and permitted minimally invasive surgery, under local anaesthesia, avoiding visible facial scarring[5,6]. Endonasal dacryocystorhinostomy (ENL-DCR) can be performed either entirely surgically[7] or with the assistance of laser to create the fistula. Massaro, Gonnering and Haris [8,9] were the first to describe the endonasal dacryocystorhinostomy (ENL-DCR), using Argon laser for the creation of the DCR fistula. Since then, carbon dioxide (CO_2_), holmium:Yag (Ho:Yag), neodymium:Yag (Nd:Yag), and potassium-titanyl-phosphate (KTP) laser systems have been employed in an attempt to identify the optimal delivery system that would achieve sufficient bone ablation with effective haemostasis[10].

Reported primary ENL-DCR success rates vary from 68% to 99% [5,6,11], depending on the type of laser, the size of the osteotomy and the use of antimetabolites, such as mitomycin C [12]. In this study, we evaluate the clinical outcome of 41 consecutive primary ENL-DCRs that were performed on 29 patients using the KTP laser over a period of one year.

## Methods

The records of all 47 ENL-DCR procedures that were performed in Freeman Hospital, Newcastle upon Tyne, United Kingdom, within a period of twelve months were retrospectively studied. The patients' main symptom was moderate to severe epiphora. Obstruction of the nasolacrimal system distal to the lacrimal sac was diagnosed with nasolacrimal syringing. Radiographic imaging was not part of the routine preoperative evaluation. Otorhinolaryngological preoperative assessment included full endoscopic examination of nasal cavities, looking for evidence of mucosal disease including polyps particularly in the middle meati. Exclusion criteria for ENL-DCR were: noticeable lower lid laxity, previous lacrimal surgery, suspicion of malignancy and previous radiation therapy. Each patient with primary nasolacrimal duct obstruction was counselled as to the advantages and disadvantages of EXT- DCR versus ENL-DCR, together with estimated success rates of the two different types of DCR. The operations were performed by the same ophthalmologist (C.N.) and ENT surgeon (S.C.). The majority of patients had surgery under local anaesthesia on an outpatient basis, except for one who opted for general anaesthesia. For local anaesthesia, Amethocaine drops were instilled in the conjunctival sac, followed by injection of Xylocaine 2% with 1:200000 Adrenaline in the medial third of both eyelids and transcaruncularly to the lacrimal sac. Cophenylcaine spray and intranasal cocaine 4% paste was applied to achieve anaesthesia and haemostasis. Dilatation of the lower punctum was performed and a 20G vitreoretinal probe was inserted in the lower canaliculus and advanced into the nasolacrimal sac. The light was directly visualised endonasally with a 0° rigid nasal endoscope, and the laser energy was delivered, with full laser precautions, via a KTP laser probe guided by the light. The nasal mucosa and lacrimal bone were ablated and the ostium was enlarged anteriorly as necessary with a microronguer. Bicanalicular O'Donoghue silicone tubes were inserted and secured with a Watzke sleeve. A course of topical Chloramphenicol drops was given for 1 week. Patients were examined 1 week postoperatively and then at 6 months for removal of tubes, or earlier, if discomfort was experienced. Mean follow up period was 16 months (range 12–24 months).

## Results

Forty- seven consecutive primary ENL-DCR operations with lacrimal intubation were performed from March 2001 to February 2002 on 35 patients with primary acquired nasolacrimal duct obstruction. Five cases with incomplete follow up and one which had to be converted to EXT-DCR due to very thick lacrimal bone were excluded from the study. Forty-one ENL-DCR procedures on 29 patients (7 males and 22 females) were included in the study. Mean age was 75 years (range 47–90, SD 13.7). 12 patients (41.4%) underwent simultaneous bilateral surgery and 17 (58.6%) had unilateral ENL-DCR. In total, 19 right sided (46.3%) and 22 left sided (53.7%) procedures were recorded (Table 1).

Pre-operatively, all patients were suffering from significant epiphora, which was affecting their quality of life. Mucocele was present in 6 cases (14.6%) and previous dacryocystitis in 4 (9.8%), while in another 3 cases (7.3%) these two conditions co-existed (Table 2). Mild medial ectropion was noted in 5 cases (12.1%) and intranasal pathology (including sinus disease, deviated nasal septum, polypoidal medial turbinate and previous nasal fractures) in 7 cases (17.1%). Two patients (4.8%) had systemic sarcoidosis.

During the operation the mean laser energy used was 400.2 joules (range 96–797, SD 188.5). Serious intra-operative complications did not occur, although in some patients' records per-operative mild discomfort was documented.

The removal of silicone tubes was scheduled at 6 months post-operatively, though in ten patients extubation was performed at 1–4 months due to discomfort. In two patients, mild nasal haemorrhage was noted on removal of tubes.

The operation was defined as being successful if the patient was asymptomatic or if there was significant improvement of symptoms, not requiring any additional procedure. Success rate at 12 months postoperatively was 78.1%. No improvement of symptoms was noted in 8 patients (19.5%), while 1 patient reported worsening of epiphora (Table 3). Pathology associated with failure (9 cases – 21.9%) included: intranasal pathology (sinus disease, septum deviation, polypoidal medial turbinate and previous nasal fracture) in 5 cases (55.5%), mucocele in 3 cases (33.3%), and systemic sarcoidosis in 1 case (11.2%) (Table 4). EXT-DCR or treatment of the nasal pathology was offered to all patients with persistent epiphora.

## Discussion

ENL-DCR is a well established surgical technique with some advantages compared to the conventional EXT-DCR. These include limitation of tissue injury to the discrete fistula site, avoidance of a skin incision, excellent haemostasis, the ability to perform a lacrimal bypass operation on an outpatient basis, quicker patient rehabilitation, decreased overall health care expense and patients' preference [8,13]. Main limitations of the technique are its steep learning curve, the higher equipment cost and its contraindication in cases of severe pre-existing nasal deformities or scarring and suspected lacrimal sac neoplasms [13].

ENL-DCR avoiding the use of laser is a well described procedure. Several surgical instruments have been employed to remove the bone overlying the lacrimal sac, including drills, osteotomes, curettes and rongeurs. Weidenbecher reported resolution or improvement of symptoms in 95% of the patients[6]. In another study of surgical ENL-DCR results, Sprekelsen achieved good results on 96% of the operations[5]. Both studies describe no major complications associated with the technique.

Various types of laser (Argon, CO_2_, Ho:Yag, Nd:Yag), have been employed in an attempt to achieve better bone ablation and haemostasis. Massaro et al [8] and Christenbury [14] reported a 70% success rate using an argon blue laser, but they both encountered difficulties in creating an adequate osteotomy. A prospective randomised comparison of EXT-DCR and ENL-DCR with the CO2-Nd:Yag laser by Hartikainen et al [15] revealed far superior results of the external approach (91% success compared to 63% with the endonasal technique), admitting though that their ENL-DCR technique was possibly suboptimal. Szubin et al [10] achieved an impressive successs rate of 97% with the Ho:Yag laser. This laser seems to outperform the rest, delivering better haemostasis and ablation, but its cost is higher and it is not so diverse in its applications [10].

The KTP laser, already utilised by ENT surgeons in other procedures, offers excellent haemostasis but its ablating properties are relatively poor, thus requiring the use of a microrongeur if the underlying bone is thick [16]. Mirza et al [17] reported improvement of symptoms in 64% of patients by KTP laser ENL-DCR, rising to 82 % including revision procedures. Using the same type of laser, Reifler found 68% success rate in a retrospective study of 19 cases, with a longer follow up of 10–16 months [18]. Interestingly, though, he noted that the first 10 cases showed a success rate of only 50%, compared to 89% in the following 9 cases. This observation reflects the steep learning curve of this technique. Mickelson et al [19] reported a series of 19 patients with 100% success (follow up 5–25 months). Hofmann et al [2] performed ENL-DCR with KTP using miniendoscopes to visualise the exact site of obstruction, and achieved success rate of 83% at one year follow up.

In our experience, the success rate of primary ENL-DCR using the KTP laser at 12 months was 78.1%. We defined success as complete resolution of epiphora or improvement of symptoms with no further procedure required, as this outcome carries the most significant implication on the patient's quality of life. The anatomic result was not evaluated at postoperative follow up, as the healed intranasal ostium size and patency do not always correlate with symptomatic relief. A significant number of patients have been reported to have symptoms in spite of a patent fistula (54% for EXT-DCR and 39% for ENL-DCR)[20], where in some cases, paradoxically, resolution of symptoms is achieved despite a negative fluorescein test [21]. As the aim of our study was to evaluate the results of primary procedures only, repeat procedures were excluded.

The optimum duration of stent retention following DCR is controversial. In published series it varies from 4 weeks to 6 months, though there is some evidence that prolonged silicone intubation may increase the incidence of DCR failure by inciting a granulomatous reaction at the internal ostium, with subsequent stenosis [22]. All our patients underwent lacrimal intubation with silicone tubes, and in most cases the tubes were removed at 6 months postoperatively. Early extubation (1–4 months) was necessary in 10 cases due to discomfort, and the operation failed in 2 of them (20%). Thus, in our experience, early removal of silicone tubes was not associated with lower success rate.

Reported pre-operative risk factors for ENL-DCR failure include pre-existing sinus disease, mucocele, nasal septum deviation, connective tissue diseases such as sarcoidosis, previous EXT-DCR, other nasal surgery, nasal fracture, and thickened lacrimal bone [23]. In our series, failure was associated mostly with intranasal pathology (sinus disease, septum deviation, polypoidal medial turbinate and previous nasal fracture), but also with mucocele and sarcoidosis (table 4). Radiographic imaging, particularly CT DCG or CT of nose and sinuses could be of potential help in clarifying the extent of concomitant sinus and nasal disease and increase our success rate. However, as this would add to the cost and the complexity of the preoperative assessment in a busy clinical setting [24], it was not part of our routine evaluation. The common outcome in the failed cases is blockage of the ostium due to cicatrisation, adhesions between the ostium and the medial turbinate, synechiae between the ostium and the septum, or granuloma formation within the ostium [22]. Opinions differ about size and location, with some surgeons favouring smaller ostium size at the lower thinner part of the lacrimal bone [25], while others recommend larger size and removal of the thicker frontal process of the maxilla [6,26]. The ideal technique is yet to be defined.

It has been proposed that the thermal energy produced by the laser may lead to scarring and subsequent blockage of the ostium [10]. It has also been suggested that the adjunctive intra-operative application of Mitomycin C (MMC) can be considered in high risk cases or primary failures, as it appears to be safe and efficient in improving the patency rate [9].

ENL-DCR performed under local anaesthesia is reported to be generally well tolerated by the patients[27]. In our study, all operations were performed under local anaesthesia, except for one patient who opted for general anaesthesia. No significant discomfort was reported by any of our patients, which confirms the reported positive patients' views. Reported complications associated with ENL-DCR include per-operative or post-operative haemorrhage, punctal erosion related to silicone intubation, silicone tubing prolapse, canalicular obstruction, orbital fat herniation, orbital and subcutaneous emphysema, conjunctival fistula formation, retrobulbar haemorrhage, and transient medial rectus paresis (23). In our series, no severe per-operative or post-operative complications were encountered. The only documented complications were discomfort caused by the silicone tubes in 10 patients requiring early extubation, and mild, transient nasal haemorrhage during removal of the silicone tubes in 2 patients.

In our experience, the use of KTP laser in ENL-DCR under local anaesthesia with the adjunctive use of an osteotome is a safe and efficient technique, with good results. The particular advantages of this laser are its superior haemostatic properties and its diversity, which reduces the cost of the operation, as it is already employed in other procedures by the ENT surgeons. Significant complications are not common with this technique. In cases of failure, revision ENL-DCR or EXT- DCR can be performed. ENL-DCR with KTP laser is routinely performed under local anaesthesia, thus avoiding the risks of general anaesthesia usually required for EXT-DCR [24] The operative time is also shorter compared to the EXT- DCR [16].

## Conclusion

ENL-DCR using KTP laser appears to be an efficient technique, with low complication rate and it is well tolerated by the patients. It still needs refinement in order to achieve the higher success rate of the EXT- DCR, which remains the gold standard method for the treatment of nasolacrimal duct obstruction. At present, we believe that patients should be involved in the decision on the type of operation, after comprehensive consultation on the advantages and disadvantages of each technique.

## Competing interests

The author(s) declare that they have no competing interests.

## Pre-publication history

The pre-publication history for this paper can be accessed here:



## Figures and Tables

**Table 1:** Characteristics of the study group

Number of patients/procedures	29/41
Mean age (years)	75
Range of age	47–90 (mean, 75)
Male:Female ratio	7:22
Laterality of surgery (right/left)	19/22
Simultaneous bilateral (patients)	12

**Table 2:** Pre-operative lacrimal drainage system abnormalities

NLD obstruction with epiphora	41 (100%)
Mucocele	6 (14.6%)
Dacryocystitis	4 (9.7%)
Mucocele and Dacryocystitis	3 (7.3%)

**Table 3:** Success rate

Asymptomatic	17 (41.5%)
Significant improvement (no further procedure required)	15 (36.6%)
Symptoms unchanged	8 (19.5%)
Symptoms deteriorated	1 (2.4%)
Total success	32 (78.1%)

**Table 4:** Pathology associated with failure

Intranasal pathology (sinus disease, septum deviation, polypoidal medial turbinate and previous nasal fracture)	5 (55.5%)
Mucocele	3 (33.3%)
Sarcoidosis	1 (11.2%)
